# Between-Quarter and Between-Position Variability of External Load Demands in a U19 Basketball Team: A 2024–2025 Hungarian Junior League Analysis

**DOI:** 10.3390/jfmk11030302

**Published:** 2026-07-30

**Authors:** Norbert Farago, David Horvath, Bence Kopper, Adam Hegedus

**Affiliations:** 1 Department of Kinesiology, Hungarian University of Sports Science, 1123 Budapest, Hungary; farago.norbi123@gmail.com (N.F.); kopper.bence@tf.hu (B.K.); 2 Basketball Academy Kecskemet, 6000 Kecskemet, Hungary; adam.hegedus.tf@gmail.com

**Keywords:** performance analysis, game period differences, team sport, technology

## Abstract

**Objectives**: External load demands (ELD) for junior basketball players have primarily been investigated in senior championships, although the competition level influences the players’ performance. The purpose of this study was to determine the between-quarter and between-position differences for U19 basketball players in their age-related championship throughout a whole competitive season. **Methods**: Data were collected from fifteen Hungarian U19 basketball players across eight home games via the WIMU PRO^TM^ local positioning system (Hudl, Nebraska, United States of America). Linear mixed models were used to evaluate omnibus main effects and position × quarter interactions for high-intensity accelerations (HIA), decelerations (HID), Player Load (PL), and vertical jumps (JUM), using Bonferroni adjustments and exploratory simple comparisons (*p* < 0.05). Moreover, model-based mean differences (MBMM) were also calculated with 95% confidence intervals (CI). **Results**: Significant game quarter omnibus main effect (*F* = 2.836, *p* = 0.043) and a borderline position × quarter interaction (*F* = 2.184, *p* = 0.050) were found in the JUM metric, with exploratory analyses revealing significant differences for centers between the second and third quarters and between the third and fourth quarters (*p* = 0.004, Mean Difference = −0.196, 95% CI = −0.345 to −0.046; *p* = 0.003, Mean Difference = 0.194, 95% CI = 0.049 to 0.338, respectively). A significant positional omnibus main effect (*F* = 4.917, *p* = 0.025) and a non-significant position × quarter interaction (*F* = 0.570, *p* = 0.753) was found for HID, with a significant difference between guards and forwards in the second quarter (*p* = 0.036, Mean Difference = 0.389, 95% CI = 0.019 to 0.742). PL and HIA did not show any significant differences. **Conclusions**: It is concluded that for the investigated sample, certain positional external load demands fluctuate between-quarter and between-position indicating that specific drills should be designed to maximize players’ performance and minimize their risk of injury. However, our results cannot be generalized due to the specificity of our dataset.

## 1. Introduction

Basketball is an intermittent sport that highly affects the players’ aerobic and anaerobic energy systems due to the game’s nature and rules [1,2]. The game is well known for high-intensity actions, including changes in direction, jumps, accelerations, decelerations, and body-to-body impacts, making them key physical factors [3,4]. Exact knowledge about the positional demands could help coaches and practitioners optimally plan and prepare their players for the game’s physical and mental demands and increase the team’s success and performance [5].

The external load demands experienced by the players are influenced by the competition level and the specific technical-tactical requirements of the different positions [6,7]. There are detailed descriptions about athletes of the same age competing in senior leagues [8,9], however the description of external load demands of Junior-age basketball players competing in their age-related championship lacks evidence.

Technological advancements have made the quantification of external load demands more objective [10]. Moreover, the increasing adoption of local positioning systems (LPS) has enabled the tracking of athletes in indoor sports environments with superior validity and reliability compared to GPS technology [11].

It is important to mention that the LPS has been accepted as a valid, reliable, and functioning athlete monitoring tool during official tournaments and games for U18 and even elite-level basketball players [7,12,13]. Quantifiable variables of interest include high-intensity actions such as high-intensity accelerations and decelerations, jumps, impacts, peak speed, change in direction, and Player Load™, a valid and reliable measurement procedure of total body workload produced by inertial microsensors [14,15,16].

Basketball players can be categorized as guards, forwards, and centers [17]. In existing literature, a wide spectrum of LPS implementations have been published, for example, comparison of different training games with real game rules and modified rules without stoppages, differentiation of external load between starters and non-starters, analyses over different positions, comparisons between junior and elite male and female basketball players [18,19,20,21]. A precise understanding of the physical demands experienced by players during competition can assist coaches and strength and conditioning professionals in tailoring training programs appropriately, helping athletes adapt to game-specific intensity levels [8,22]. Recent studies have examined variability among basketball player positional and game quarter demands during official junior-aged male tournaments in external load metrics such as total covered distance, high-intensity accelerations (≥2 m·s^−2^) and decelerations (≤−2 m·s^−2^) [14]. Other researchers have analyzed official Spanish second league home matches throughout a complete season, using variables like Player Load™, Peak Speed, jumps higher than 3-G forces and impacts over 8 G-forces, while other studies used intensive changes in direction like the total amount of movements registered in the rightward and leftward vector even in the high-band zone (>3 m·s^−2^) [8,13]. Previous studies have determined external load variables across 5 friendly games using principal component analysis (PCA), which minimizes the overlap between the observed variables to reduce redundancy [15,23]. In previous research, the authors managed to define the specific external load metrics for all basketball-related positions, for example, changes in direction and deceleration for guards and forwards, while jumps and deceleration for centers, highlighting that external load monitoring in basketball should emphasize the most informative variables describing positional demands [15].

There are published differences in some physical abilities, like aerobic and anaerobic power, jumping ability, and anthropometric measures between the abovementioned basketball positions [24,25]. Positional variability in external load metrics has been published in the existing literature measuring U18 male basketball players, resulting in significant differences in relative covered distance, high-intensity accelerations and decelerations, peak velocity and jumps [7,8]. There is consensus in the literature that guards tend to achieve the most high-intensity accelerations and decelerations, while reaching the highest values in peak acceleration and covered total distance [7,8]. In contrast, centers complete the smallest amount of high-intensity accelerations and decelerations and covered distance per minute; interestingly, in some studies they have performed the most high-speed running with the highest number of vertical jumps [7,8]. There is a high fluctuation concerning forward external load parameters, while they mix the roles of the guards and centers and must participate in every game phase like offense, defense, and transition [15,26,27]. The distinctive characteristics of the forward position have been documented in the literature. While some studies have shown that forwards cover the highest relative distances and accumulate the greatest number of total and high-intensity decelerations, other investigations have reported contrasting findings, indicating that forwards perform the fewest high-intensity accelerations and decelerations among all playing positions [6,8,28].

Numerous studies have found significant differences in external load demands, such as total covered distance, high-intensity accelerations, decelerations, and Player Load™ between the first and last quarter even in male and female U18 basketball players during official tournaments [8,14,26,29]. By examining every quarter individually, researchers have found significant differences in Player Load^TM^/min and changes in direction in the first and second quarters between backcourt (i.e., guards) and frontcourt players (i.e., forwards and centers) [9]. Also, this study mentioned that in the third and fourth quarters, the frontcourt players showed a significantly higher number of accelerations compared to other findings where authors addressed that centers executed a significantly higher number of jumps in the last quarter in comparison to the other two positions [9,30]. However, limited research is available for junior basketball players for a whole competitive season where players have participated in their own age-related championship.

Due to the positional roles, variations can also be observed between them within a given quarter. A study analyzing the youth women’s basketball final four tournament has found position-related patterns, where guards tend to increase their workload until the end of the game with less variability in the external load metrics, in contrast with the forward whose performance dropped in the fourth quarter [26]. In another study, which included every in-season game and examined Chinese professional basketball athletes, the authors found significant differences between guards and centers during the first quarter in Player Load^TM^/min and during the fourth quarter in the number of high-intensity jumps and high-intensity accelerations. A significant difference has been found during the first quarter between guards and forwards in the performed medium-intensity jumps [30]. Such findings can be seen in collegiate Chinese basketball players, where the authors have found significant differences between backcourt players (guards) and frontcourt players (centers and forwards) during the first three quarters in Player Load^TM^/min, however, the backcourt players performed significantly fewer accelerations than their frontcourt counterparts in the last three quarters [9].

In the existing literature, some authors have examined the variability of external demands with players of similar chronological age; however, these players have participated in a senior second league [8,9]. There is limited research available examining the external load fluctuation of Junior-age (U19) basketball players through a whole competitive season competing in their age-specific league, leaving a gap in these players’ external demand description. Relying on findings derived from Junior-age players competing in senior leagues could cause inaccurate conditioning and preparation strategies, as the external load demands of these players could differ from those of players who play in their age-related championship.

The current study addresses this problem, providing a description of external load demand fluctuations in a U19 basketball team participating in the Hungarian Junior League. The aim of our research is (1) to determine the fluctuation in external load variables between quarters (between-quarter) for a given position, and (2) to uncover the difference between positions within a pre-defined quarter (between-position). Accordingly, it is hypothesized that (1) external load demands will decrease significantly as the game progresses between-quarter and (2) significant differences will be observed between-position.

## 2. Materials and Methods

### 2.1. Participants

A total of 15 basketball players (age: 17.6 ± 1.17 years; height: 1.89 ± 0.08 m; body mass: 79.3 ± 9.78 kg) participated in the study. The athletes played in the same team, divided into three positions: guards (*n* = 7), forwards (*n* = 4), and centers (*n* = 4). All players belonged to the Junior team of a Hungarian basketball academy and had been playing since U14 age. The participants competed only in the domestic championship for the 2024/25 season, as approved by the Hungarian Basketball Federation. The players participated in 5–6 basketball practices weekly with 1 or 2 strength workouts besides their weekly game. The head coach and assistant coach consented to participation. All players had to sign a declaration of consent. For players who had reached the age of 16 but had not yet reached the age of 18, the signature of their parent or legal guardian was also required. Ethical approval was granted by the Ethics Committee of the Hungarian University of Sports Science (document number: MTSE-OKE-KEB/01/2023) and adhered to the guidelines and ethical principles outlined in the Declaration of Helsinki [31].

### 2.2. Design

The research was organized to define the variability in external load demands of a Hungarian Junior basketball team during official competitions. Fifteen U19 basketball players were monitored throughout every home game from 6 October 2024 to 9 April 2025 during the 2024/25 season. The team participated in 16 official games during the entire season, with 8 home games, and all eight home games were included in the final analysis, resulting in *n* = 271 player-quarter observations. No overtime periods occurred during these matches. One player was excluded entirely from the study for not contributing to any games during the season.

**Figure 1:**
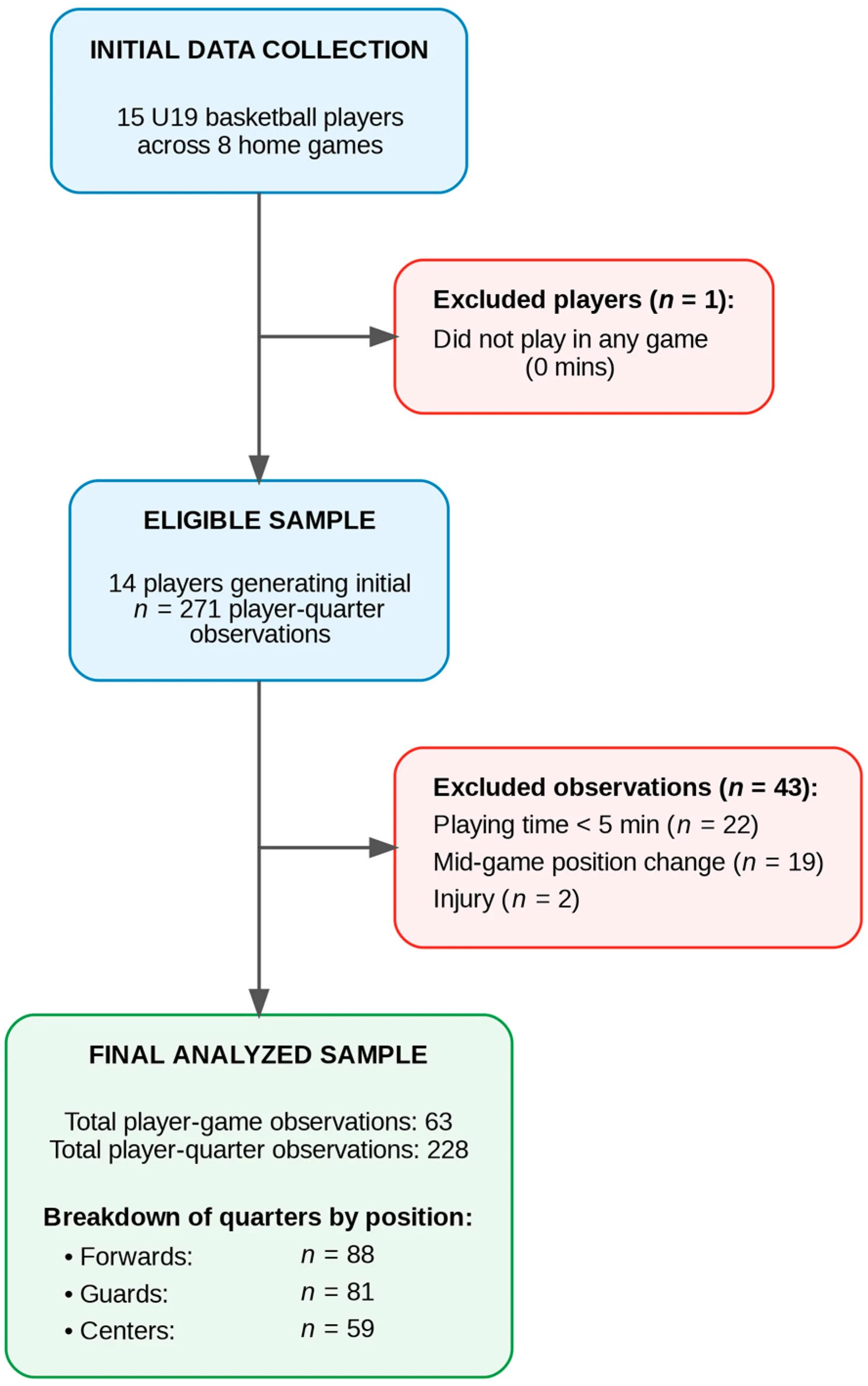
Participant flow diagram of data processing. Blue boxes represent the initial data collection and eligible sample, the green box indicates the final analyzed sample, and red boxes detail the excluded players and observations. Arrows indicate the flow of the sample selection process.

Exclusion criteria were applied based on established methodology [11]. Specifically, observations were excluded if a player sustained an injury (*n* = 2 observations removed) or failed to reach a minimum total playing time of 5 min per match (*n* = 22 observations removed) due to substitutions or fouling out with five personal fouls, thereby eliminating potential noise and distorted load estimations originating from minimal match exposure. Consequently, out of the 14 monitored players, an additional 3 players were completely excluded from the final analyzed sample because they failed to meet this 5 min playing time requirement in any of the games. Furthermore, to ensure that each player remained in the same positional category throughout the study, any observation where a player temporarily changed positions mid-game was excluded (*n* = 19 observations). The participant flow diagram of data processing is presented in Figure 1.

**Figure 2:**
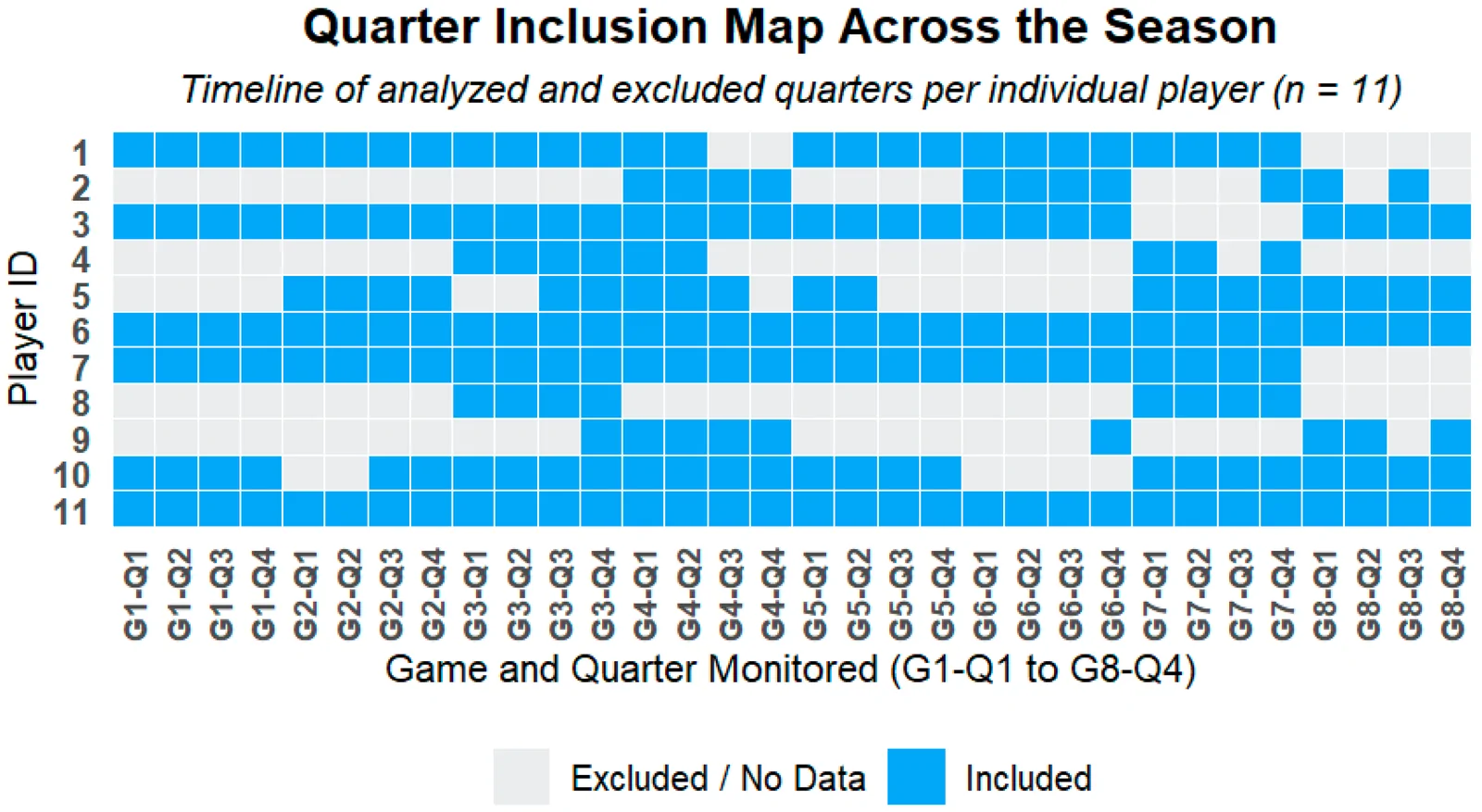
Players’ quarterly participation in each game.

Following these exclusion criteria, a final sample of *n* = 11 players, contributing to *n* = 63 player-game observations and *n* = 228 player-quarter observations, was included in the final analysis. The players’ exact quarterly participation for each individual game is visualized on the heatmap in Figure 2.

The exact number of observations contributed by each individual player is detailed in Supplementary Table S1. The final sample breakdown by playing position was *n* = 88 for forwards, *n* = 59 for centers, and *n* = 81 for guards.

### 2.3. Methodology

The games were organized and completed on the same court with FIBA-approved dimensions (28 m × 15 m) in consistent environmental conditions. Physical preparation was consistent prior to all 8 home games with a standardized 30 min warm-up protocol under the supervision of the team’s strength and conditioning coach. The protocol followed a fixed progression, divided into three 10 min phases: (1) dynamic mobilization and activation exercises, (2) technical drills, including dribbling, shooting, and 2-versus-3 situational exercises performed at match-specific intensities, and (3) shooting practice, specifically free throws and three-pointers. This structured approach ensured that the nature and intensity of the preparation remained consistent throughout the competitive season. The players were allowed to drink water ad libitum.

The games consisted of four 10-min quarters, interspersed with 2 min of rest between the first and last two quarters. The half-times were separated by a 15-min break. All players were monitored during warm-ups and matches using the local positioning system detailed in Section 2.4. External load parameters were recorded exclusively during active playing time. Data collection commenced the moment a player entered the court and was automatically paused upon substitution, repeating each time the player re-entered the game [32]. The required time for free throw execution was included in the total time. The rest time between quarters, half-times, and the official time-outs was not included in the measurement. Overtime periods were excluded from the final time calculation. Quarters without playing time did not generate any observations.

The collected results from each game were interpreted through temporal and positional segmentation approaches. Each game was divided into four discrete quarters (Q1, Q2, Q3, Q4), allowing for the analysis of between-quarter and between-position variability. Each player-quarter combination was treated as an individual data point if they met the inclusion criteria (minimum five minutes of gameplay, no mid-game positional changes, and no injury during any part of the game).

To calculate the between-quarter and between-position differences, the external load variables were grouped by playing positions and quarters.

If the system experienced any recording interruptions, we discarded the data from that given match. During the observation period, we did not experience any signs of recording interruptions.

### 2.4. External Load Parameters

The external load parameters were recorded via the WIMU PRO^TM^ local positioning system (Hudl, Lincoln, NE, USA). The sensor (81 mm × 45 mm × 16 mm, 70 g) includes four 3D accelerometers, a gyroscope, a 3D magnetometer with 100 Hz sample frequency, a GPS with 10 Hz sample frequency, and an ultra-wideband positioning system with 18 Hz sample frequency. Each device has a microprocessor, 8 GB flash memory plus a high-speed USB plug for other data management procedures, like storing, recording and data uploading. The inertial units were held in a harness during the recording in a vest recommended by the manufacturer (IMAX, Lleida, Spain), located on the upper back between the scapulae. During the data collection, each participant wore the same size vest according to their anthropometric characteristics to ensure the right fit for the individuals.

The external load parameters recorded, similarly to other studies included (a) Total Distance Covered (TDC), (b) total distance covered with acceleration higher than 1.12 m·s^−2^ (EXD), (c) High Intensity Accelerations (≥2 m·s^−2^) (HIA); and Decelerations (≤−2 m·s^−2^) (HID)); (d) distance covered above 18 km·h^−1^ (HSRD), (e) Player Load shown in arbitrary units and calculated as the sum of square rates of change in acceleration in the three vectors divided by 100 (PL), (f) the number of jumps > 3 G (JUM), (g) the number of impacts > 8 G (IMP), (h) Dynamic Stress Load, the sum of weighted acceleration-based impacts higher than 2 G (DSL), and (i) High Metabolic Load Distance, distance covered above 25.5 W·kg^−1^ Metabolic Power (HML) [7,8,14,33,34,35]. All the above-mentioned variables have been normalized to played minutes in accordance with the earlier mentioned methodology [36].

Although multiple external load metrics were monitored during the games, four primary parameters were selected for advanced statistical modeling using Linear Mixed Model (LMM). In accordance with the available literature, metrics like HIA, HID, JUM and PL were selected for deeper analysis [8,13,15].

Previous research proved that the WIMU PRO^TM^ (Hudl, Lincoln, NE, USA) system has high accuracy for monitoring positional data and has good inter-unit reliability, having better accuracy during distance and mean velocity measurement than GPS [12,37]. There were 12 antennas installed around the field. The antennas were positioned at the following distances from the center of the game field: 2 antennas at 20.7 m, 2 at 15.7 m, 2 at 17.7 m, 2 at 16.5 m, 1 at 17.5 m, 1 at 21 m, 1 at 19 m, and 1 at 21.5 m. The registered data were downloaded and analyzed using the SPRO^TM^ 2.3.1 (Hudl, Lincoln, NE, USA) software provided by the manufacturer.

### 2.5. Statistical Analysis

An a priori sample size calculation was conducted using G*Power (version: 3.1.9.7, University of Dusseldorf, Dusseldorf Germany) [38]. The calculation was performed using an F-test family (ANOVA: Repeated measures, within-between interaction) with a power of 0.95 and an alpha error level of 0.05. Based on the pre-defined alpha level, *n* = 45 was determined as the required sample size.

Before the analysis, the assumption of normality was checked using the Q-Q plot and the Shapiro–Wilk’s W test. The differences in external load demands were assessed with a linear mixed model (LMM). The external load demands were treated as dependent variables, the players’ position (guards, forwards and centers) and the quarters (first, second, third and fourth quarter) as fixed effects, while the players’ ID was treated as a random effect.

Primary fixed effects and interaction terms were evaluated using Type III Tests of Fixed Effects. The significance of the omnibus main effects for position and quarter, as well as their interaction, were assessed prior to conducting pairwise comparisons. To control the Type I error inflation due to multiple comparisons, Bonferroni post hoc correction was applied in pairwise comparisons. In the absence of a statistically significant position*quarter interaction, subsequent pairwise comparisons were treated as exploratory simple comparisons. The statistical significance was set at *p* < 0.05. All data were analyzed using SPSS statistical software (31.0.2.0 Version for Windows, SPSS, IBM, Armonk, NY, USA). The descriptive statistics from the championship games were reported as mean ± standard deviation (SD), while the results of the selected external load demands from the linear mixed model were reported as model-based mean differences (MBMM) with 95% confidence intervals (CI) for greater transparency.

## 3. Results

The descriptive statistics as mean and standard deviation for the external load parameters not included for statistical analysis are shown in Table 1.

The descriptive statistics of the external load demands included for statistical analysis are presented in Table 2 as mean and standard deviation. Complete numerical model outputs for these selected parameters are provided in the Supplementary Material as Supplementary Tables S2–S4.

**Figure 3:**
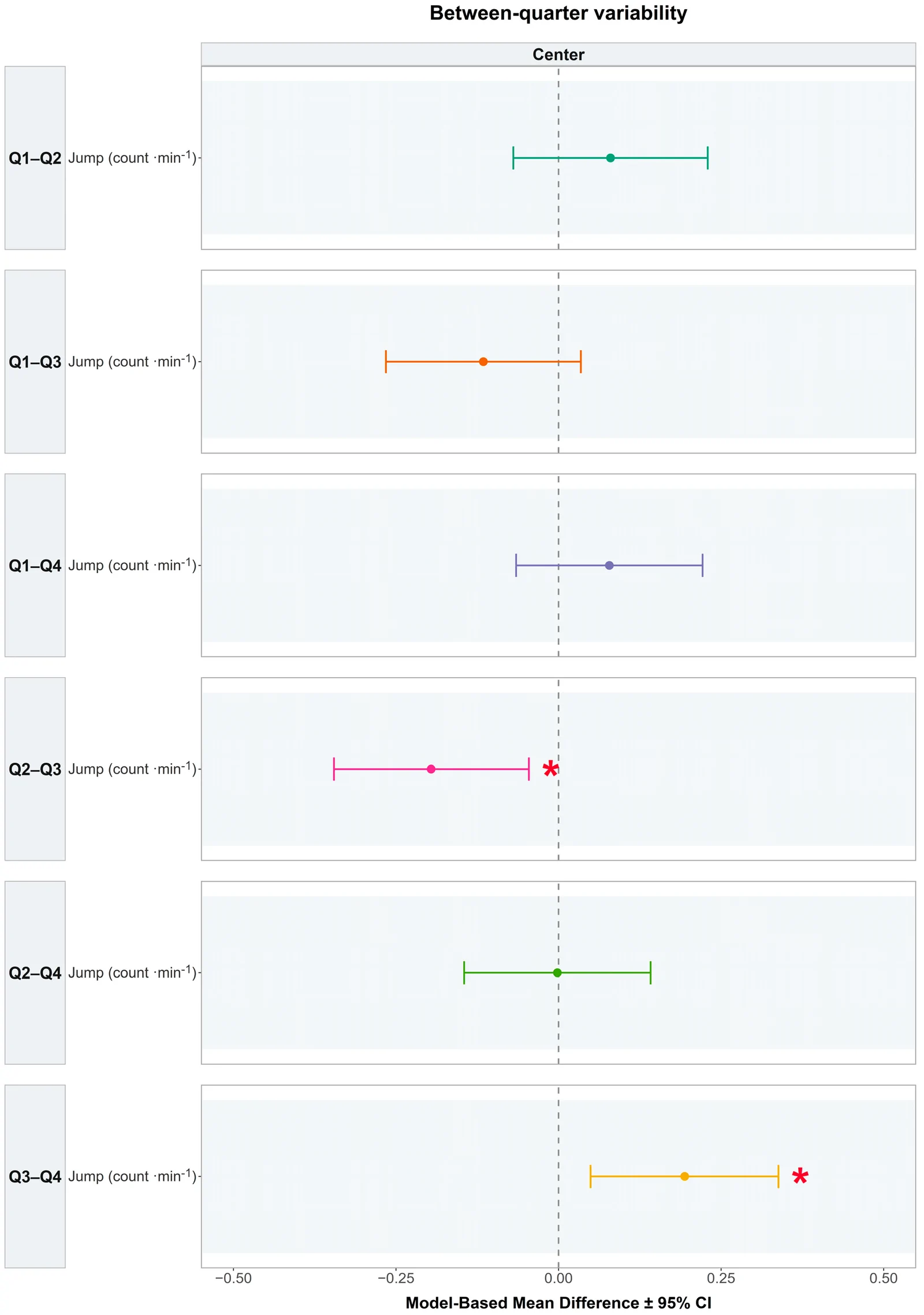
Exploratory between-quarter variability in the Jump (JUM) metric for the center position. Data are presented as model-based mean differences ± 95% confidence intervals (CI). The direction of the pairwise comparisons is established as the earlier quarter minus the later quarter (e.g., Q1 vs. Q4 is calculated asQ1–Q4). Consequently, a positive value indicates a higher jump output in the earlier quarter, whereas a negative value signifies a higher jump output in the later quarter. Asterisks (*) denote a statistically significant difference (*p* < 0.05). Abbreviations: Q1, first quarter; Q2, second quarter; Q3, third quarter; Q4, fourth quarter; min, minute; CI, confidence interval; JUMP, Jumps > 3 G.

While analyzing the between-quarter fluctuation, omnibus analysis revealed a significant main effect of game quarter (F = 2.836, *p* = 0.043) in the JUM variable, while the main effect of playing position was not significant (F = 0.261, *p* = 0.772). The analysis of the position × quarter interaction presented a *p*-value on the threshold of statistical significance (Numerator df = 6, Denominator df = 103.310, F = 2.184, *p* = 0.050). Given the sample size, this borderline result cannot be definitively classified as statistically significant; however, it indicates a tendency toward a position-specific quarter pattern. Based on this meaningful trend, we conducted exploratory simple comparisons, which revealed significant differences for centers between Q2–Q3 and Q3–Q4 (*p* = 0.004, Mean Difference = −0.196, 95% CI = −0.345 to −0.046; *p* = 0.003, Mean Difference = 0.194, 95% CI = 0.049 to 0.338, respectively). The results in JUM for centers are presented in Figure 3.

**Figure 4:**
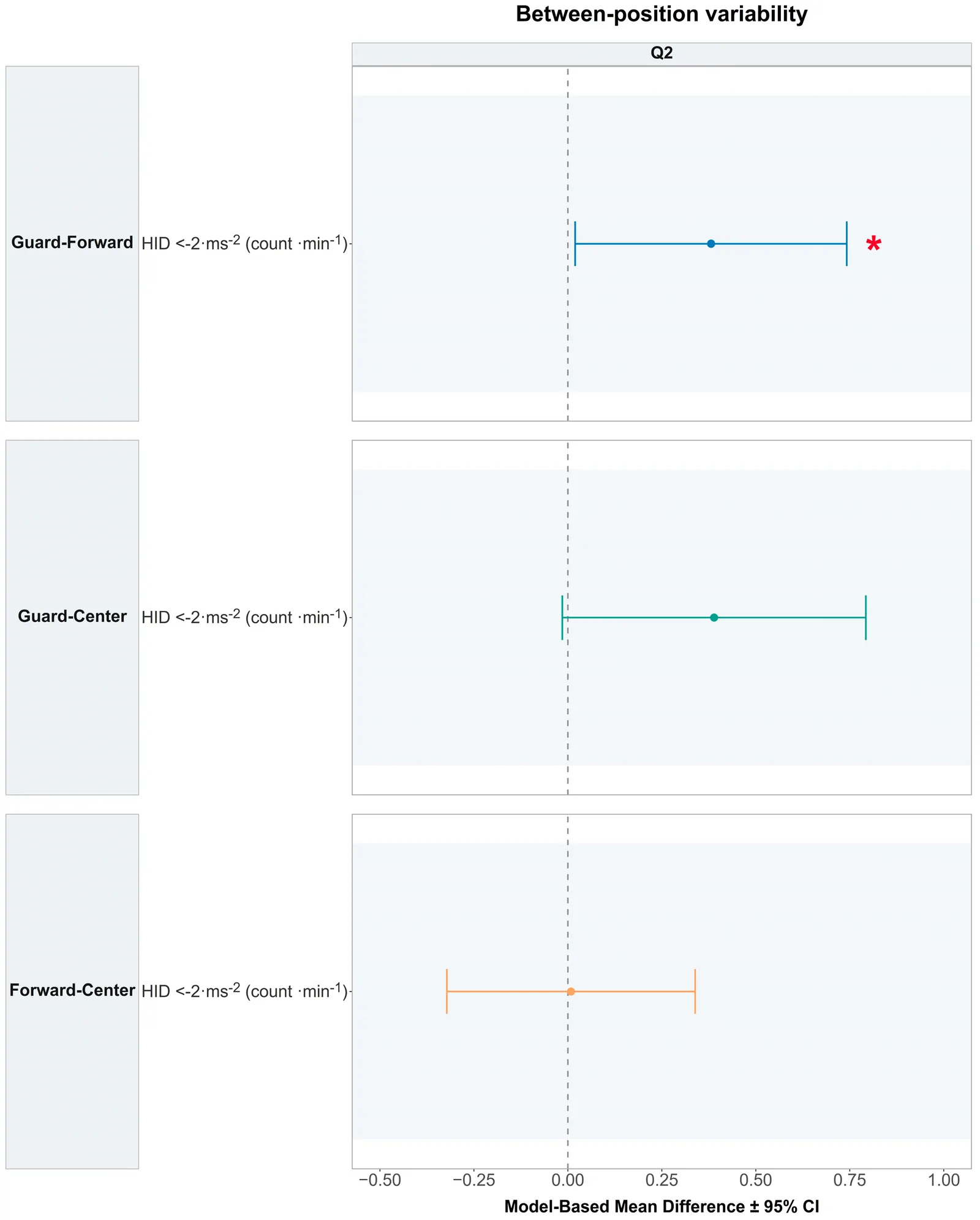
Exploratory between-position variability in the High-Intensity Deceleration (HID) metric during the second quarter (Q2). Data are presented as model-based mean differences ± 95% confidence intervals (CI). The direction of the pairwise comparisons is established as the first-named position minus the second position (e.g., Guard minus Center). Consequently, a positive value indicates a higher deceleration output for the former position, whereas a negative value signifies a higher output for the latter position. Asterisks (*) denote a statistically significant difference (*p* < 0.05). Abbreviations: CI, confidence interval; HID, High-Intensity Deceleration; min, minute; Q2, second quarter.

During the examination of between-position variability in the above-mentioned selected variables, the linear mixed model omnibus analysis revealed a significant positional main effect for HID (F = 4.917, *p* = 0.025), whereas the effect of game quarter was not significant (F = 1.661, *p* = 0.181). Importantly, the position × quarter interaction was also not statistically significant (Numerator df = 6, Denominator df = 70.23, F = 0.570, *p* = 0.753). Due to the lack of significant position*quarter interaction, further positional analyses within specific quarters were conducted as exploratory simple comparisons. Based on this exploratory approach, post hoc Bonferroni pairwise comparison showed significant differences between guards and forwards during Q2 (*p* = 0.036, Mean Difference = 0.389, 95% CI = 0.019 to 0.742). The exploratory between-position comparison for HID during Q2 is illustrated in Figure 4.

For the remaining variables (PL and HIA), we found no significant main effect in the omnibus analysis; therefore, we did not conduct any further analysis.

## 4. Discussion

The main objectives of this study were to identify the variability in external load demands between-quarter for a given position and between-players (between-position) for a given quarter regarding a fixed position. Following the statistical analysis, our results indicated distinct variations in some of the selected metrics, reinforcing the deviation between the players’ roles in different tactical aspects of the game. These differences were confined to highly specific, position-dependent tactical metrics. Based on our exploratory simple comparisons, we observed distinct patterns, particularly for High-Intensity Decelerations (HID) for guards during the second quarter, and in vertical jumps (JUM) for centers during the second half of the game.

Main findings of the current study showed an overall decline in every external load demand, without significant differences between the first and last quarters. In agreement with previous findings, except JUM, the highest values for all examined variables were recorded in the first quarter, likely reflecting the teams’ effort to establish an early advantage and adapt to their opponents’ strategies [11]. Moreover, an exploratory analysis indicated a positional difference in the HID metric during Q2, where guards demonstrated significantly higher values compared to forwards. Additionally, exploratory simple comparisons showed differences in the JUM metric for centers between Q2–Q3 and Q3–Q4.

Centers executed the most vertical jumps with a unique pattern. This could occur because they usually position themselves under the basket based on tactical aspects like rebounding and actions near the basket. [13,28,39]. In the third quarter, the vertical movement activity of the centers increased, making the greatest number of vertical jumps during the game; however, we did not find any significant difference between the positions in the mentioned quarter [30,40]. The former described increment in vertical jumps is already mentioned in the literature, explaining the lack of uncertain and unnecessary shots performed by the perimeter players, preferring actions closer to the basket and explaining the number of contacts they accomplish in the area close to the basket [11,40]. Our results further contribute to the aforementioned literature. Statistically, our LMM revealed a significant omnibus main effect for the game quarter (*p* = 0.043) regarding the JUM variable. While the position × quarter interaction was exactly at the threshold of statistical significance (*p* = 0.050), the data revealed a meaningful trend in positional differences across quarters. Based on the significant main effect and this interaction tendency, our subsequent exploratory simple comparisons revealed distinct differences for centers, particularly a significant increase between Q2 and Q3 (*p* = 0.004), followed by a significant decrease between Q3 and Q4 (*p* = 0.003). While we must be cautious in interpretation, this noticeable trend provides a strong foundation for future investigations. The significant increase in the number of vertical jumps performed by centers in Q3 can be explained by proper recovery during the half-time rest, accomplished by adequate lactate clearance and glycogen resynthesis [41]. A similar increase in external load metrics in Q3 has also been mentioned in the literature, where the authors examined female youth basketball players during a final tournament [26]. The significant decrease between Q3 and Q4 can be explained by localized neuromuscular fatigue accumulation due to explosive movements [8,40]. These findings support earlier research explaining that tactical and technical adjustments, together with changes in players’ roles, may influence external load demands as the game progresses [7,8,40]. The aforementioned results of our research support our first hypothesis, as significant differences were found during the between-quarter analysis, due to the significant main effect of quarter in the JUM variable.

Analyzing between-position variability, LMM revealed a significant omnibus main effect for the playing position (*p* = 0.025) regarding the HID metric. In the absence of a statistically significant position × quarter interaction (*p* = 0.753), subsequent exploratory simple comparisons indicated a significant difference in HID between guards and forwards (*p* = 0.036) exclusively during Q2 in favor of guards. This position tends to perform the most changes in direction as a result of their responsibility of making quick actions during events of the gameplay [7,14,28,42]. This can be explained by their specific positional demand, like their playmaking role to create the optimal situation and space to occupy shots [25,43]. Our results are partially confirmed based on the available research, examining such aspects of between-position variability, where significant differences were observed in the frequency of changes in direction during the first and second quarter for guards [29]. In our study, we could not find any significant differences in the number of jumps, Player Load and intensive accelerations between-position, while there is a distinct increase based on our results in the number of jumps executed by the centers during the third quarter with a significant difference between Q2 and Q3. While other publications found significant differences in performed jumps higher than 40 cm in the last game period, direct comparison is limited due to different measurement systems and age groups [29,30].

Concerning the forwards, as noted above, we found a significant between-player difference during Q2 via exploratory analysis, where they performed significantly fewer high-intensity decelerations than guards (*p* = 0.036). Regarding the other analyzed variables, the forwards’ performance remained stable throughout the games. This phenomenon can be explained by the forwards’ specific role in modern basketball, while they participate in transitions, ball and off-ball screens, and also in free space to occupy easy shots [28,43,44].

The above-mentioned findings support our second hypothesis, while we found significant differences during the between-position analysis.

Regarding the remaining variables, both PL and HIA showed descriptive decreases toward the end of the game. However, based on our statistical model, no significant differences were found in either between-position or between-quarter analyses. This lack of statistical significance is in direct agreement with other findings, where the authors also did not report any significant differences when examining between-position and between-quarter variations in player workload [8].

Accepting the above detailed information, the study also has some limitations. We could not monitor internal load, which could have given a more precise description of the positional demands and their variability. We could only measure the home games due to the fixed setup of the local positioning system. Also, we could only monitor one team, consisting of 15 under-19 basketball players, meaning a relatively low sample size.

The limited sample size in our study could explain the lack of significant position ×  quarter interactions in certain external load metrics. Consequently, our findings cannot be broadly generalized and should be interpreted with caution. We would suggest further analysis, involving basketball players from different age groups and female basketball players also for a comprehensive analysis. Also, further investigation is needed to describe the variability in external load variables, including internal load and away game measurements, to precisely define them in the players’ age-specific championship.

## 5. Conclusions

This research investigated the between-quarter and between-position variability of external load demands in a U19 basketball team during their age-specific championship. The investigated sample demonstrated distinct variability in the external load demands with detected position-dependent variations between-quarter and between-position.

Our study demonstrated that these specific workload variances across different quarters highlight the highly specialized tactical-technical roles of each playing position. Exploratory analysis indicated that guards performed significantly more high-intensity decelerations than forwards, especially during the second quarter, reflecting their explosive playmaking role. Centers executed an increased number of vertical jumps after the half-time break. The significant increase in vertical jumps was followed by a significant decrease in the last quarter, possibly due to fatigue accumulation. Conversely, the forwards successfully fulfilled their versatile, hybrid role without any significant difference between-quarter and between-position.

In conclusion, the specific nature of this external load variability suggests that coaches and strength and conditioning professionals should limit the application of universal training protocols applied to the entire team. Our study demonstrated that a position’s external load profile is complex, depending heavily on both the specific game quarter and the tactical demands. However, our results cannot be widely generalized due to the limited sample size of the athletes participating in the competition.

Based on our results, individually tailored and specific training programs may be required to elicit the desired adaptations for every position, focusing on eccentric strength for guards, vertical power for centers, and well-rounded conditioning with multidirectional explosive movement for forwards. In addition, our results can assist coaches to plan and control specific drills to mimic the game’s demand to maximize on-court performance while minimizing injury risk.

## Figures and Tables

**Table 1:** Descriptive statistics normalized for minutes played for the external load demands not included for statistical analysis.

Position	Quarter	TDC (m·min^−1^)	EXD (m·min^−1^)	HSR (m·min^−1^)	IMP (Count·min^−1^)	DSL (a.u.·min^−1^)	HML (m·min^−1^)
Guard (*n* = 7)	Q1	70.97 ± 21.13	10.38 ± 3.08	0.66 ± 0.74	0.98 ± 0.68	3.71 ± 2.26	17.87 ± 6.14
	Q2	57.55 ± 19.55	7.43 ± 3.08	0.43 ± 0.74	0.88 ± 0.51	3.22 ± 1.81	12.25 ± 4.85
	Q3	69.09 ± 12.50	9.05 ± 2.27	0.44 ± 0.55	0.97 ± 0.63	3.39 ± 1.64	14.85 ± 4.00
	Q4	62.59 ± 15.65	8.36 ± 3.39	0.31 ± 0.47	1.00 ± 0.66	3.71 ± 2.18	13.15 ± 5.35
Center (*n* = 4)	Q1	69.50 ± 17.88	10.07 ± 3.15	0.74 ± 1.14	1.73 ± 1.47	5.66 ± 3.91	17.54 ± 5.92
	Q2	54.80 ± 21.54	6.78 ± 3.71	0.11 ± 0.33	1.21 ± 1.28	4.51 ± 3.21	11.79 ± 7.20
	Q3	64.95 ± 18.38	9.18 ± 3.25	0.21 ± 0.40	1.57 ± 0.75	5.14 ± 2.29	15.81 ± 6.21
	Q4	61.62 ± 17.61	8.11 ± 2.96	0.09 ± 0.39	1.65 ± 1.55	4.62 ± 3.14	12.98 ± 5.12
Forward (*n* = 4)	Q1	67.59 ± 17.73	10.05 ± 3.43	1.02 ± 1.11	1.24 ± 1.01	3.88 ± 2.46	18.31 ± 6.69
	Q2	57.81 ± 17.15	8.22 ± 3.18	0.64 ± 0.98	0.97 ± 0.82	3.28 ± 1.90	14.09 ± 5.92
	Q3	68.42 ± 10.77	9.66 ± 2.39	0.73 ± 1.00	1.23 ± 0.86	3.81 ± 2.09	16.90 ± 5.39
	Q4	59.83 ± 13.48	8.01 ± 2.27	0.42 ± 0.58	1.00 ± 0.82	3.36 ± 2.05	13.72 ± 4.20

Abbreviations: TDC (m·min^−1^), Total Distance Covered per minute; EXD m·min^−1^, Explosive Distance, distance covered with acceleration higher than 1.12 m·s^−2^ per minute; HSRD (m·min^−1^), High Speed Running above 18 km·h^−1^ per minute; IMP (count·min^−1^), Impacts > 8 G per minute; DSL (a.u.·min^−1^), Dynamic Stress Load, the sum of weighted acceleration-based impacts higher than 2G per minute; HML (m·min^−1^), High Metabolic Load Distance, distance covered above 25.5 W·kg^−1^ Metabolic Power per minute; Q1, first quarter; Q2, second quarter; Q3, third quarter; Q4, fourth quarter; m, meter; min, minute.

**Table 2:** Descriptive statistics normalized for played minutes for the external load demands included for statistical analysis.

Position	Quarter	HIA (Count·min^−1^)	HID (Count·min^−1^)	PL (a.u.·min^−1^)	JUM (Count·min^−1^)
Guard (*n* = 7)	Q1	0.76 ± 0.37	0.87 ± 0.38	1.29 ± 0.39	0.13 ± 0.13
	Q2	0.64 ± 0.39	0.58 ± 0.29	1.03 ± 0.41	0.12 ± 0.12
	Q3	0.80 ± 0.27	0.86 ± 0.35	1.21 ± 0.28	0.15 ± 0.11
	Q4	0.71 ± 0.37	0.78 ± 0.44	1.12 ± 0.34	0.17 ± 0.2
Center (*n* = 4)	Q1	0.88 ± 0.32	0.67 ± 0.36	1.34 ± 0.33	0.31 ± 0.25
	Q2	0.60 ± 0.35	0.44 ± 0.28	1.06 ± 0.38	0.23 ± 0.24
	Q3	0.90 ± 0.48	0.52 ± 0.24	1.24 ± 0.29	0.43 ± 0.22
	Q4	0.71 ± 0.45	0.45 ± 0.38	1.19 ± 0.39	0.21 ± 0.23
Forward (*n* = 4)	Q1	0.79 ± 0.53	0.62 ± 0.52	1.21 ± 0.34	0.20 ± 0.20
	Q2	0.58 ± 0.24	0.43 ± 0.25	1.04 ± 0.31	0.13 ± 0.13
	Q3	0.69 ± 0.40	0.55 ± 0.20	1.20 ± 0.24	0.17 ± 0.13
	Q4	0.57 ± 0.25	0.43 ± 0.25	1.04 ± 0.27	0.14 ± 0.11

Abbreviations: HIA (count·min^−1^), High Intensity Acceleration (≥2 m·s^−2^) per minute; HID (count·min^−1^), High Intensity Deceleration (≤−2 m·s^−2^) per minute; PL (a.u.·min^−1^), Player Load, the sum of square rates of change in acceleration in the three vectors divided by 100 per minute; a.u., arbitrary unit; JUM (count·min^−1^), Jumps > 3 G per minute; Q1, first quarter; Q2, second quarter; Q3, third quarter; Q4, fourth quarter; m, meter; min, minute.

## Data Availability

The data presented in this study are available on request from the corresponding author. The data are not publicly available due to ethical reasons.

## Supplementary Materials

The following supporting information can be downloaded at: https://www.mdpi.com/article/10.3390/jfmk11030302/s1, Table S1: Individual player participation data; Table S2: Type III Tests of Fixed Effects; Table S3: Pairwise Comparisons Between-Position; Table S4: Pairwise Comparisons-Between-Quarter.

## Abbreviations

The following abbreviations are used in this manuscript:

ELD	External load demands
U19	Under nineteen
GPS	Global Positioning System
LPS	Local Positioning System
PCA	Principal Component Analysis
MIN	Minute
3D	Three dimensions
Hz	Hertz
GB	Gigabyte
USB	Universal Serial Bus
TDC	Total Distance Covered
EXD	Explosive Distance
HIA	High Intensity Acceleration
HID	High Intensity Deceleration
HSR	High Speed Running
PL	Player Load
JUM	Jump
IMP	Impact
DSL	Dynamic Stress Load
HML	High Metabolic Load Distance
LMM	Linear Mixed Model
SD	Standard Deviation
MBMM	Model-based mean difference
CI	Confidence Interval
ID	Identification
Q1	First quarter
Q2	Second quarter
Q3	Third quarter
Q4	Fourth quarter
